# Self-Nitrogen-Doped Nanoporous Carbons Derived from Poly(1,5-diaminonaphthalene) for the Removal of Toxic Dye Pollutants from Wastewater: Non-Linear Isotherm and Kinetic Analysis

**DOI:** 10.3390/polym12112563

**Published:** 2020-10-31

**Authors:** Ali Aldalbahi, Badr M. Thamer, Mostafizur Rahaman, Mohamed H. El-Newehy

**Affiliations:** Department of Chemistry, College of Science, King Saud University, Riyadh 11451, Saudi Arabia; bthamer@ksu.edu.sa (B.M.T.); mrahaman1997@gmail.com (M.R.); melnewehy@ksu.edu.sa (M.H.E.-N.)

**Keywords:** polymer materials, nitrogen doping, nanoporous carbon, wastewater treatment, adsorbent, adsorption, dye removal

## Abstract

The high surface area and porosity of self-nitrogen-doped porous carbons (SNPCs) nominates them for potential application in water treatment due to their high efficiency towards the removal of various pollutants. In this study, SNPCs were fabricated from poly(1,5-diaminonaphthalene) (P(1,5-DANPh) by single and simultaneous carbonization at the activation step at different temperatures (600, 700, and 800 °C). The carbonization’s temperature plays a vital role in controlling the nitrogen-doping, surface area, porosity, and morphology of SNPCs. The SNPCs-7 sample prepared at 700 °C showed the highest surface area (1678.8 m^2^ g^−1^) with pore volume (0.943 cm^3^ g^−1^) with a micro/meso porous structure. The prepared SNPCs were used as an effective adsorbent for removal of crystal violet dye (CV) from contaminated water. SNPCs-7 showed the highest adsorption of 487.53 mg g^−1^ and the adsorption capacity of the SNPCs samples follows the order SNPCs-7 > SNPCs-8 > SNPCs-6, which is consistent with the results of their surface area and porosity. The adsorption for CV dye followed Freundlich isotherm models and a pseudo second order kinetic model. The negative values of Gipps free energy (Δ*G*°) and positive value of enthalpy (Δ*H*°) indicated that the adsorption of CV dye onto the surface of SNPCs was a spontaneous and endothermic process, respectively. Based on the results, the adsorption mechanism of CV dye onto the surface of SNPCs was proposed.

## 1. Introduction

The problem of providing potable and agricultural water is one of the most challenging that facing water-poor countries. Moreover, the problems of water scarcity and pollution of water that affect many countries encourage the researchers to develop new materials to overcome these problems [1,2]. The treatment and reuse of wastewater is one of the effective solutions to mitigate the problem of water scarcity and to protect the environment from pollution [3,4]. The industrial sector is one of the largest sectors that consumes a large amount of water due to most of its processes dependent on water [5]. Moreover, most of the water used in industrial processes is not completely consumed and is discharged wastewater that contains various pollutants. Among these industries that use large quantities of water are textile, cosmetics, printing, and the tanning industry, and the reuse of their wastewater will save water and reduce the environmental pollution [6]. To reuse wastewater, its treatment is necessary to eliminate pollutants such as dyes, which are the most dangerous one present in industrial wastewater with high toxicity and stability even at low concentrations [7]. Various methods such as coagulation [8], precipitation [9], photodegradation [10], biodegradation [11], and adsorption [12] were used for dyes removal from wastewater. The choice of the appropriate method for removing pollutants from aqueous systems depends on the cost, efficiency, and the non-production of secondary pollutants, and the adsorption technique can meet these requirements [13,14]. The adsorption efficiency as well as the cost depend mainly on the properties of the adsorbent and its reusability. There are many adsorbent materials that have been used, such as polymers [15], metal–organic frameworks (MOFs) [16], carbonaceous materials [17] and clays [18]. Carbon-based materials are the most common and effective adsorbents due to their low cost and variety of sources. However, the adsorption efficiency of the pristine and bulk carbon-based materials to remove pollutants was below the desired level. Therefore, the efficiency of pristine and bulk carbon-based materials with respect to removing various pollutants can be improved via creating functional groups and pores on the surface [19,20].

Porous carbon-based materials (PCMs) with their pore size range from a micro- to meso-structure have been proven to remove dyes and others pollutants from water due to their high specific surface area, high pore volume, and high stability in acidic and alkaline media [21]. PCMs can be produced from both biopolymers [22] and synthetic polymers [23]. PCMs can be prepared via three routes, including carbonization followed by activation, template, and in situ activation. However, the carbonization followed by activation process involved two steps and the high cost of templates process restrict their practical applications. Therefore, the in-situ activation method is a preferred method as the carbonization and activation process can be done synchronously in one step with low cost [24]. On the other hand, the production of carbon materials with a high yield remains one of the challenges facing researchers due to the breakdown of organic materials and polymers during the carbonization and activation process in the form of gases. Polyacrylonitrile (PAN) is one of the best polymers used in the production of carbon materials with a high yield of more than 50% [25]. Recently, due to the high cost of PAN and its need for stabilization before carbonization and activation process, there is a need to find an alternative source such as conjugated polymers like polyaniline, polypyrrole, and poly(phenylenediamine). These conjugated polymers can be used for the preparation of self-nitrogen doped porous carbons (SNPCs) by high temperature carbonization in the presence of activating agents [26,27,28]. For example, SNPCs were prepared by the carbonization of polyaniline at high temperature in the presence an activating agent and showed a good efficiency towards the removal of pollutants [29].

In the present study, self-nitrogen doped porous carbons (SNPCs) with a high surface area were prepared from poly(1,5-naphthalinediamine) (P(1,5-DANPh)) by a facile and single step process using activating agent such as KOH at different temperature. In this synthesis route, carbonization, activation, and self-nitrogen doping occur simultaneously. The as-prepared SNPCS were characterized by different techniques and exhibit an excellent adsorption capacity towards the removal of CV dye as a model of cationic dyes with the possibility of reusability for more than five times.

## 2. Materials and Methods

### 2.1. Materials

Briefly, 1,5-Diaminnaphthalene was obtained from BDH Chemical. Ammonium persulfate, acetonitrile, hydrochloric acid, potassium hydroxide, sodium hydroxide, and CV dye were obtained from Sigma–Aldrich Co. (St. Louis, MO, USA) Supplementary Figure S1 shows the chemical and molecular 3d structure of CV dye.

### 2.2. Preparation of Poly(1,5-diaminonaphthalene) (P(1,5-DANPh))

For the typical synthesis of polymer, in a round-bottomed flask, 23.7 g of 1,5-diaminonaphthalene dissolved in 375 mL of acetonitrile was purged at 25 °C with nitrogen for 15 min. In a 200-mL of volumetric flask, ammonium persulfate (41.7 g) dissolved in distilled water was purged with nitrogen gas. After that, the ammonium persulfate solution was added to 1,5-diaminonphthaline dropwise over 40 min. Then, the reaction was subject to constant stirring at 25 °C for 24 h. The black precipitate, poly(1,5-naphthalinediamine) P(1,5-DANPh), was separated by filtration, washed several times with distilled water, and dried at 60 °C in vacuum oven overnight.

### 2.3. Preparation of Self-Nitrogen-Doped Porous Carbons (SNPCs)

P(1,5-DANPh) and KOH were ground in a ratio of 1:4 by a regular blender and then the mixture was placed directly in vacuum oven overnight. The carbonization and activation processes were carried out semitonally in furnace tube for one hour at various temperatures (600, 700, and 800 °C) under nitrogen with temperature rate of 5 °C min^−1^. After cooling the furnace to RT, the product was collected and dispersed in 1M of HCl with stirring for 2 h at 50 °C followed by filtration and washing several times with distillated water until neutral pH for the filtrate. The product (SNPCs) was dried in a vacuum oven overnight at 80 °C. The prepared samples are referred to as SNPCs-6, SNPCs-7 and SNPCs-8.

### 2.4. Characterization Self-Nitrogen-Doped Porous Carbons (SNPCs)

The prepared P(1,5-DANPh) and the corresponding SNPCs samples were characterized by routine techniques. Field emission scanning electron microscopy with energy dispersive X-ray (FESEM-EDX, JEOL2100F, Tokyo, Japan) spectroscopy was used to study the morphology and elemental analysis. The structure of polymer and the surface functional groups of SNPCs was identified by Fourier-transform infrared spectroscopy (FTIR, Thermo Fisher Scientific, Waltham, MA, USA) in the range of 400 to 4000 cm^−1^ using a KBr pellet. The crystallinity and graphitization degree of P(1,5-DANPh) and SNPCs were studied by X-ray diffraction (XRD, MiniFlex, Rigaku, Tokyo, Japan). The textural characteristics (e.g., pore volume, pore size and surface area) of the P(1,5-DANPh) and SNPCs was evaluated by using Brunauer–Emmett–Teller analysis (BET, Micromeritics-ASAP-2020, Micromeritics, Norcross, GA, USA) conducted via N_2_ adsorption at 77 K. The thermal behavior of P(1,5-DANPh) and SNPCs was studied using thermogravimetric analysis (TGA, Q500-USA, Canberra, Australia). Zeta potential values were measured at different pH values and carried out by Zeta potential (Nano Plus zet/nano, Gerbrunn, Germany) through the dispersion of 10 mg of adsorbent in 10 mL distillated water by sonication for 5 min at 25 °C before measurement.

### 2.5. Adsorption Study

The adsorption of CV dye onto SNPCs was studied in batches via many parameters, including the effect of pH, ionic strength, initial concentration of CV dye, contact time, as well as temperature. The adsorption experiments were conducted in 20-mL polypropylene tubes. To 10 mL of dye solution at pH 10, the adsorbent (10 mg) was added and shaken using a thermostat shaker water bath at a speed of 70 rpm for 4 h. Then, the tubes were put in a tube holder for 15 min to settle the adsorbent, and the supernatant was collected for the measurement of unadsorbed CV dye. The residual concentration of CV dye was measured using a UV/Vis spectrophotometer (Perkin-Elmer, Lambda 35, Buckinghamshire, UK) at λ_max_ = 589 nm. The effect of pH on the adsorption of CV dye was conducted at pH 3 (acidic), pH 7 (neutral), and pH 10 (alkaline). All media were adjusted by 0.1 mol L^−1^ of HCl or NaOH. The effect of ionic strength was performed at different concentration of NaCl (0.1, 0.3 and 0.5 mol L^−1^) at pH 10, 500 mg L^−1^ of CV dye and adsorbent dosage 10 mg. The effect of initial concentration of CV dye was studied by carrying out the adsorption experiments in the concentration range between 100 and 800 mg L^−1^ at room temperature.

Kinetic studies were done by adding 10 mg of the adsorbents was to 10 mL of CV dye solution (300 mg L^−1^) under agitation at 25 °C. The concentration of the residual CV dye was followed by measuring the absorbance at regular time intervals by taken 100 μL of dye solution which was diluted to 3 mL with distilled water.

The efficiency of the adsorbent and the amount of adsorbed CV dye onto the adsorbents at equilibrium (*q*_e_, mg g^−1^) and time *t* (*q*_t_, mg g^−1^) were calculated using the following Equations (1)–(3):

(1) $$q_{t}=\frac{C_{o}-\;C_{t}}{m}\times \;V$$

(2) $$q_{e}=\frac{C_{o}-\;C_{e}}{m}\times \;V$$

(3) $$\%\;Removal\;efficiency\;=\frac{C_{o}-\;C_{e}}{Co}\times 100$$

where *C_o_*, *C_t_*, and *C_e_* (mg L^−1^) are the initial, *t* time, and equilibrium concentrations of the dye solution, respectively. V (L) is the volume of dye solution and m (g) is the adsorbents mass.

### 2.6. Desorption Study

The adsorption-desorption of CV dye was conducted in 20-mL polypropylene tubes by adding 10 mg of the adsorbent to 10 mL of CV dye (100 mg L^−1^) and placing on a thermostat shaker water bath at a speed of 70 rpm for 2 h. The tube was placed on the holder for 15 min to settle out the adsorbent and the supernatant solution was completely taken and the absorbance was measured. The desorption process was carried out by adding 10 mL of ethanol and 100 μL of HCl (0.1 mol L^−1^) as desorption agent to the adsorbent, shaking for 5 min, then let to settle the adsorbent. Finally, the adsorbent was left to settle out and the absorbance of the desorbed solution of dye was measured as mentioned earlier. The adsorption–desorption process was repeated for five cycles.

### 2.7. Error Analysis

The nonlinear regression method is a reliable tool to define the best fitting experimental data of adsorption and kinetic process. To determine the best kinetic and isotherm model for representing experimental data, Chi-square values (χ^2^) and coefficient of determination values (*R*^2^) were calculated for the nonlinear method by Equations (4)–(6). The model with the lowest value of χ^2^ and the highest value of *R*^2^ is the most representative of the experimental data and the least error.

(4) $$\chi_{red}^{2}=\sum_{i}^{n}\frac{{\left(q_{e,exp}-q_{e,model}\right)}^{2}}{n_{p}-p}$$

(5) $$R^{2}=1-\;\frac{\sum {\left(q_{e,exp}-q_{e,model}\right)}^{2}}{\sum {\left(q_{e,exp}-q_{e,mean}\right)}^{2}}$$

(6) $$R_{red}^{2}=1-\left(1-\;R^{2}\right)\cdot \;\left(\frac{n_{p}-p}{n_{p}-p-1}\right)$$

where *q*_e,*model*_ is each value of *q_e_* predicted by the fitted model, (*q*_e,*exp*_) is each value of *q*_e_ measured experimentally, *q*_e,*mean*_ is the average value of *q*_e_ that was measured experimentally, *n_p_* is the number of performed experiments, and *p* is the number of parameters for the fitted model.

## 3. Results & Discussion

### 3.1. Characterization of Self-Nitrogen-Doped Porous Carbons (SNPCs)

#### 3.1.1. Morphology of (SNPCs)

**Figure 1:**
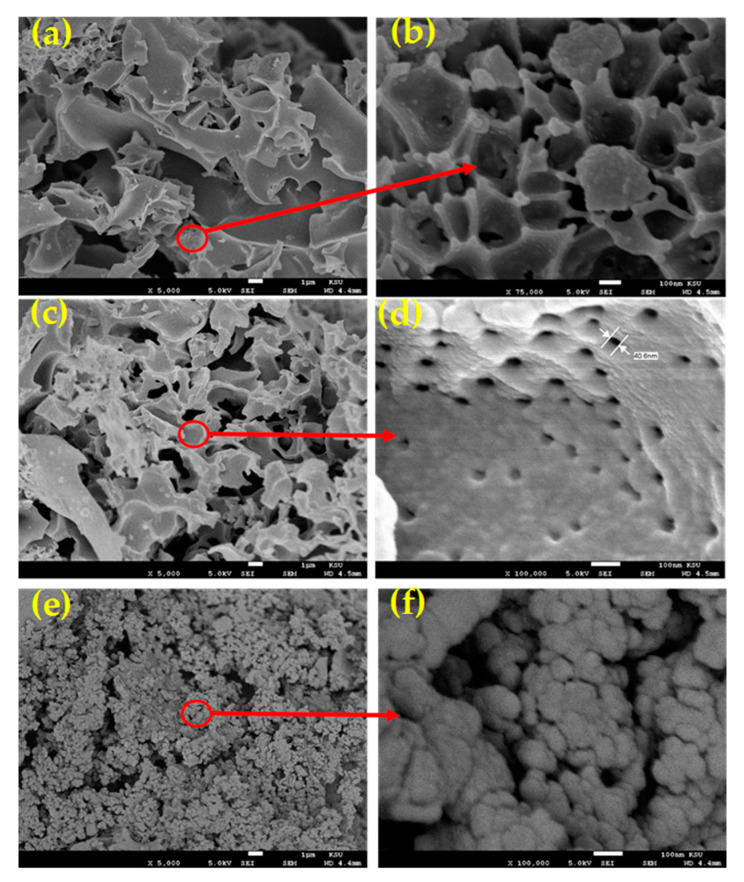
Low and high-resolution FESEM images of (**a**,**b**) SNPCs-6, (**c**,**d**) SNPCs-7 and (**e**,**f**) SNPCs-8.

Figure 1 displays the low and high-resolution FESEM images of SNPCs that was prepared at three-different temperatures. Figure 1a and b showed that the SNPCS-6 sample has flake shape and macroporous structure with open channels. Upon increasing the temperature to 700 °C, the flake shape changed to a herringbone shape and connected pore channels with a lesser size increased with a uniform distribution and porous structure, as shown in Figure 1c,d. The high and uniform porosity of SNPCS-7 is attributed to deeper etching arising from increasing the temperature. However, the increase in temperature to 800 °C led to the agglomeration and the conversion of the flake shape to spherical form accompanied with the damage of the interconnected channels and mesoporous structure in SNPCS-8, as shown in Figure 1e,f. The morphology of SNPCs after adsorption CV dye was also investigated, as shown in Supplementary Figure S4. After adsorption dye, the surface of SNPCs and pores became more smother, implying that CV dye molecules had attached to the SNPCs surface.

#### 3.1.2. BET Analysis

**Figure 2:**
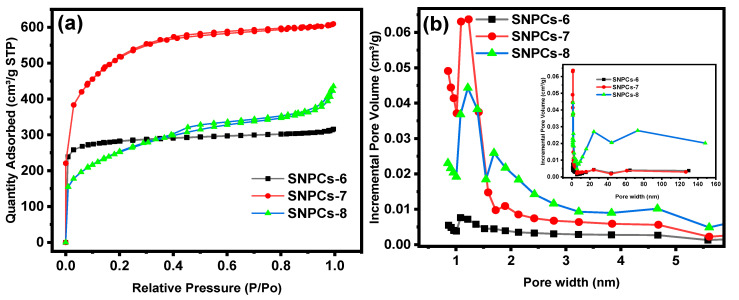
(**a**) N_2_ adsorption-desorption isotherms and (**b**) pore size distribution for SNPCs.

Isotherms of nitrogen adsorption–desorption and the pore size distributions are displayed in Figure 2 and the textural characteristic properties of SNPCs are listed in Table 1. SNPCs samples show Type I isotherm and amount of adsorbed nitrogen increased rapidly at pressure less than 0.2. This result confirms a high affinity between adsorbent and adsorbate; and the adsorbent possess micropores structure. However, samples SNPCs-7 and SNPCs-8 also exhibit Type IV isotherm which indicted to also them possess a mesoporous structure. The mesoporous ratio of the SNPCs samples increase with the temperature of carbonization, and were 26.38%, 74.70%, and 91.98 % for SNPCs-6, SNPCs-7, and SNPCs-8, respectively. The average of pore width of SNPCs-6, SNPCs-7, and SNPCs-8 was 2.28, 2.25, and 3.1 nm, respectively. Interestingly, the SNPCs-7 exhibits the highest surface area (1678.8 m^2^ g^−1^) with pore volume (0.943 cm^3^ g^−1^). The order of the surface area and pore volume followed SNPCs-7 > SNPCs-8 > SNPCs-6. Based on the FESEM and BET results, it can be concluded that the temperature of the carbonization activation process plays an essential role in creating porous structures and the optimum temperature is 700 °C for the preparation of self-nitrogen doped porous carbons, which are characterized by high surface area and micro/meso porous structure. Similarly, Tian et al. have found the optimized temperature of carbonization to produce porous carbon from cellulose with a high surface area was 700 °C [30].

#### 3.1.3. FTIR Spectra

**Figure 3:**
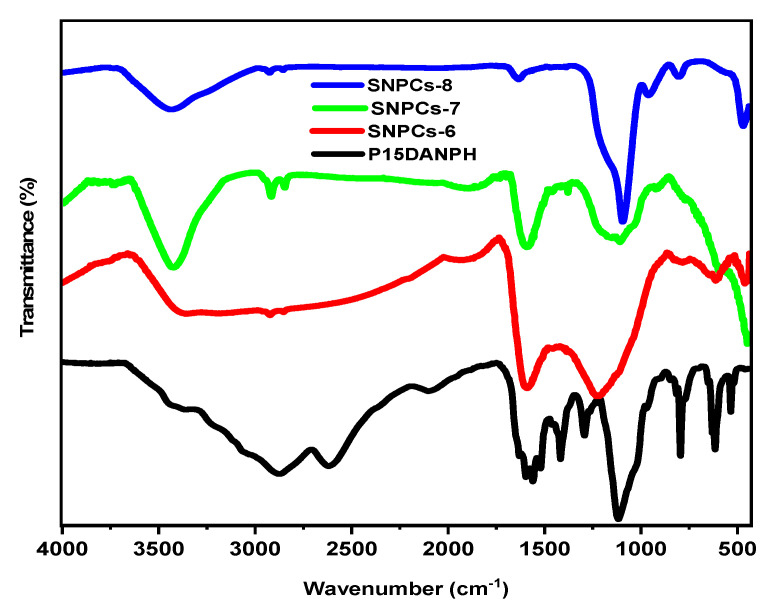
FTIR spectrum of P(1,5-DANPh) and SNPCs.

Figure 3 shows the FTIR spectra of P(1,5-DANPh) and SNPCs. The FTIR spectrum of P(1,5-DANPh) showed multiple peaks in the range of 400–4000 cm^−1^ and most of them disappeared after the carbonization activation process. The FTIR spectrum of P(1,5-DANPh) showed multiple peaks at 3358, 2873, 1630, 1596–1417, 1293, 1117, and 815–518 cm^−1^, which are related to N–H stretching, -C=NH+- stretching, C=N stretching, C=C stretching, C–N stretching for primary amine, C–C inter-ring, and C–H out-of-plane, respectively [31]. Most of its peaks disappeared after the carbonization activation process. This result indicates that the polymer is completely converted to SNPCs product. The sample of SNPCs-6 has only two characteristic peaks at 1601 and 1227 cm^−1^, which were attributed to C=C and C–N stretching vibration, respectively. These results indicated the success of the carbonization process and the conversion of P(1,5-DANPh) into nitrogen-doped carbon structure, while the activation process is incomplete. As the carbonization activation temperature was increased, new characteristic peaks for SNPCs-7 and SNPCs-8 appeared at 3437 and 1113 cm^−1^; and at 3436, 958, 796, and 465 cm^−1^, respectively. These were attributed to the presence of oxygen-containing functional groups that were formed on the surface of SNPCs by the activation process using KOH, which is more effective at both 700 and 800 °C.

#### 3.1.4. EDX Analysis

**Figure 4:**
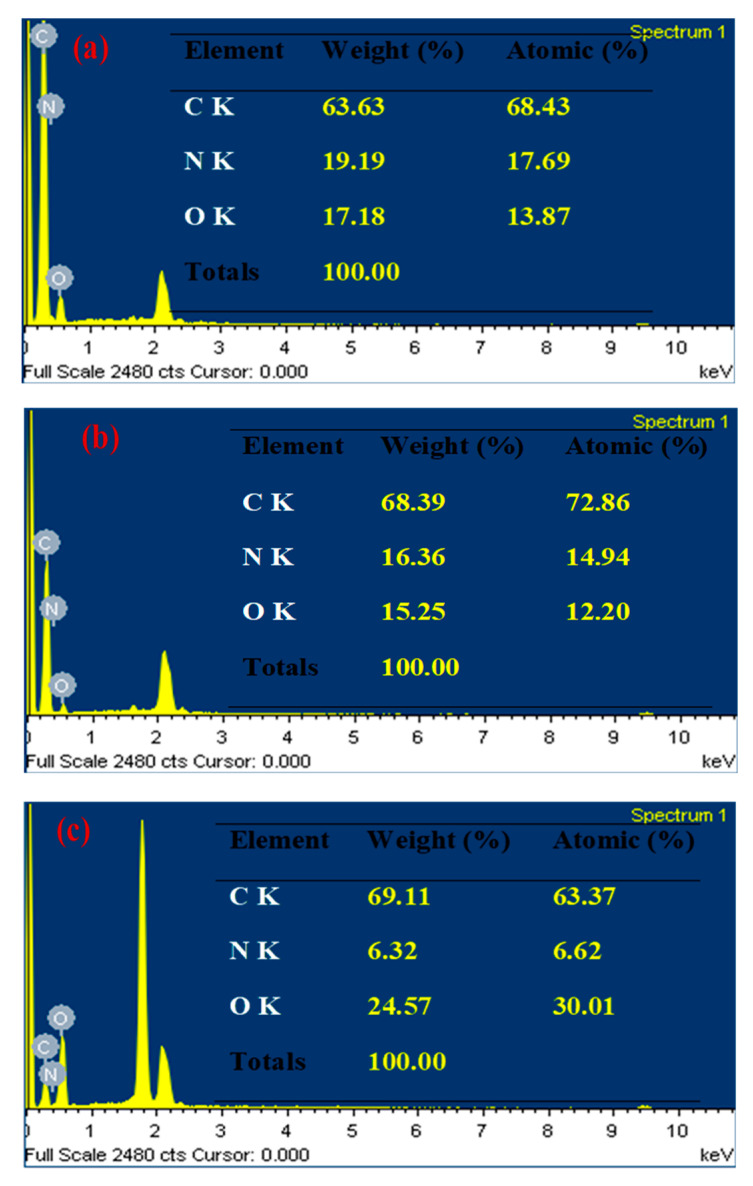
EDX analysis of (**a**) SNPCS-6, (**b**) SNPCS-7 and (**c**) SNPCS-8.

The EDX analysis of SNPCs samples confirmed the presence of the elements; carbon, nitrogen and oxygen (Figure 4). The amount of the doped nitrogen depends mainly on the temperature of calcination-activation process, and it was found to be 17.69%, 14.94%, and 6.62% for SNPCS-6, SNPCS-7, and SNPCS-8, respectively. The obtained results confirmed the high self-nitrogen doping and the creation of oxygen-containing functional groups on the surface of the porous carbon structure obtained by the carbonization activation process.

#### 3.1.5. XRD Analysis

**Figure 5:**
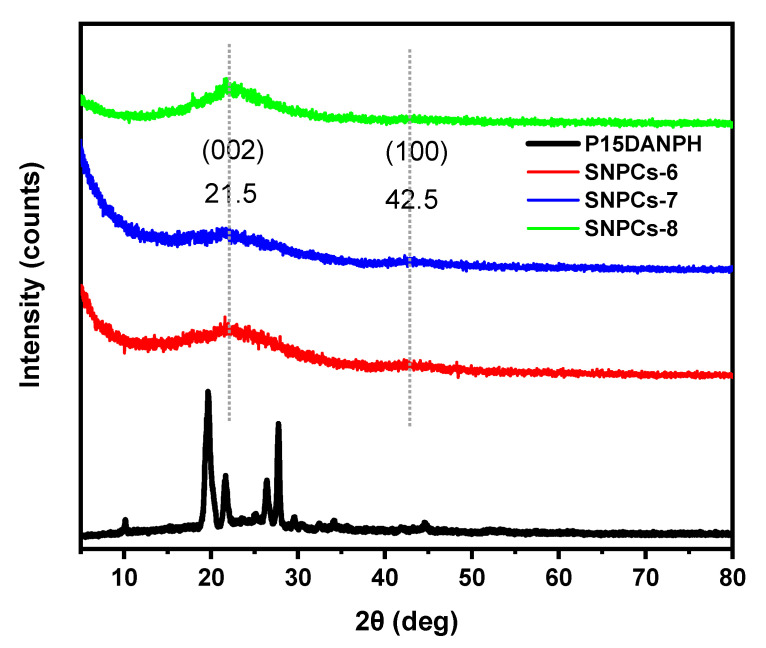
XRD analysis of P(1,5-DANPh), SNPCS-6, SNPCS-7 and SNPCS-8.

Figure 5 displays the XRD analysis of P(1,5-DANPh) and SNPCs samples. As shown in Figure 5, P(1,5-DANPh) showed sharp diffraction peaks that reflect its high degree of crystallinity. During the carbonization activation process, most diffraction peaks of polymer disappeared and SNPCs showed only two broad peaks at 21.6° and 42.5° due to the conversion of the polymer to the disordered graphite structure and a slight ratio of transformation to the graphite composition, respectively. The interlayer spacing of SNPCS-6, SNPCS-7, and SNPCS-8 was 0.411, 0.425 nm, and 0.398 nm, respectively, which is bigger than that of graphite (0.335 nm). This result indicated that SNPCs samples have the highest ratio of C–C(*sp*^3^) bond and more disordered structure due to the formation of oxygen-containing functional groups during the activation process [32].

#### 3.1.6. Thermogravimetric Analysis

**Figure 6:**
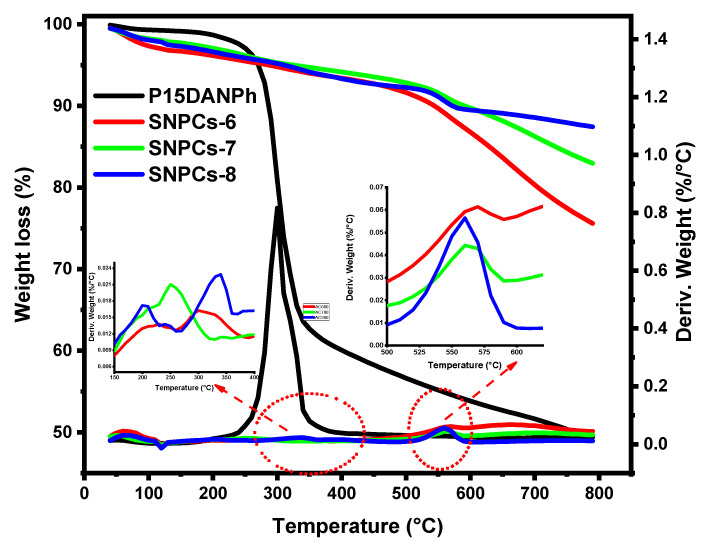
TGA/DTG thermogram of P(1,5-DANPh) and SNPCs.

The thermal decomposition of P(1,5-DANPh) and samples was studied by TGA-DTA as shown in Figure 6. The thermogram of P(1,5-DANPh) showed that the degradation takes place in single step in the range 230–270 °C, peaked at 300 °C (*T_max_*) and leaving residue of 49.39% at 800 °C. The high percentage of residue reflects the importance of P(1,5-DANPh) as a promising polymer for the preparation of self-nitrogen-doped porous carbon materials. It noteworthy that the thermal stability of SNPCs depends on the temperature of carbonization-activation process as can be seen in Figure 6. SNPCs showed good thermal stability and the residual at 800 °C was found to be 75.59, 82.96, and 87.44 °C for SNPCs-6, SNPCs-7, and SNPCs-8, respectively. Three steps of thermal decomposition for SNPCs samples took place, at which the first step was assigned to the decomposition of less stable oxygen containing functional groups such as carboxylic groups resulting from the activation process at 200–400 °C. The second step was assigned to the decomposition of more stable oxygen containing functional groups such as hydroxyl groups at 500–600 °C. The third step is attributed to the partial collapse of the carbon structure and the liberation of doped nitrogen as nitrogen gas over 600 °C.

### 3.2. Adsorption Study

#### 3.2.1. Effect of pH

**Figure 7:**
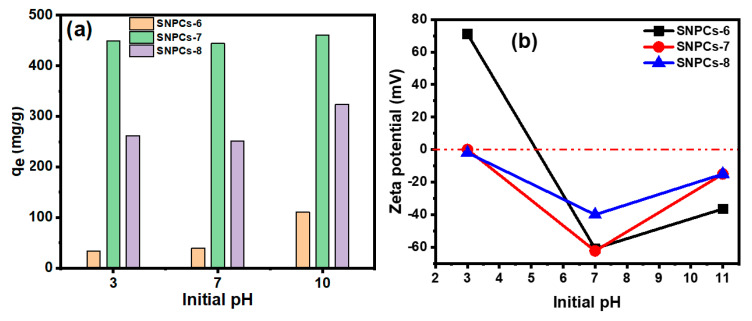
(**a**) pH’s effect on the adsorption of CV dye onto adsorbents (SNPCs) and (**b**) zeta potential vs initial pH of SNPCs dispersed in distillated water.

pH plays an important role in the adsorption process of pollutants, especially for ionic pollutants, in functionalized carbon materials. This was assigned to the change in electrostatic attraction between the ionic dye molecules and the surface of adsorbent. Therefore, pH effect on the adsorption capacity of CV dye onto the SNPCs-6, SNPCs-7 and SNPCs-8 was studied at acidic, neutral and alkaline media at 25 °C and CV concentration of 500 ppm. Figure 7 showed that the adsorption capacity of CV dye onto all adsorbents has no noticeable change in both acidic (pH 3) and neutral (pH 7) media but in alkaline medium (pH 10) there is a significant increase in the adsorption capacity of SNPCs-6 and SNPCs-8. In alkaline medium, the ionization of oxygen-containing groups increased, resulting in the formation of an anionic surface of SNPCs, which can be more suitable for the adsorption of cationic CV dye. Due to the p*K_a_* value of phenolic groups being approximately 8.0–9.0, the surface charge of SNPCs the SNPCS-6, SNPCS-7, and SNPCS-8 will become predominantly negative at pH higher than the p*K_a_*. From the obtained results, the performance of SNPCs-6 and SNPCs-8 as adsorbents for CV dye was improved in alkaline media while SNPCS-7 is an effective adsorbent in all pH’s. These results indicated the important role of pores@SNPCs-7 in the adsorption process across the diffusion of dye through it. The zeta potential vs. initial pH of SNPCs was studied as shown in Figure 7b. The charge on the surface of SNPCs-7, SNPCs-7, and SNPCs-8 is correlated with pH values of solution and they exhibit a negative charge at pH 7 and 11. This result agrees with that result of pH’s effect on adsorption capacity.

#### 3.2.2. Effect of Ionic Strength

**Figure 8:**
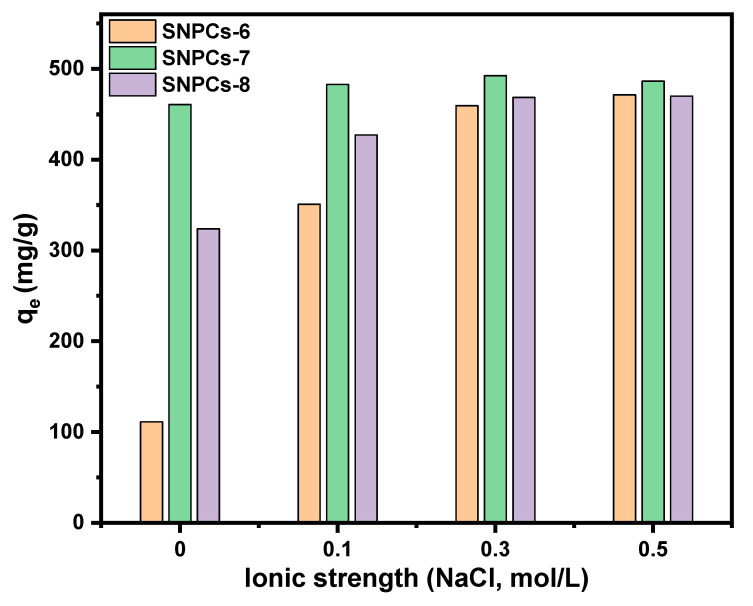
Ionic strength effect on adsorption of CV dye onto the prepared adsorbents (SNPCs).

The ionic strength effect on adsorption of CV dye onto SNPCs can assist the interpretation of the adsorption mechanism. Figure 8 showed that the increase in the concentration of NaCl results is accompanied with an increase in the adsorption capacity of CV dye onto SNPCs-6 and SNPCs-8 compared to slightly increase onto SNPCs-7. With increasing the concentration of NaCl from 0 to 0.3 mol L^−1^, the adsorption capacity was increased from 111.21, 460.77, 323.86 to 459.37, 492.58, and 468.68 mg g^−1^ for SNPCs-6, SNPCs-7, and SNPCs-8, respectively. This increase in adsorption capacity onto SNPCs is due to the decrease in interactions between CV dye and water molecules due to the presence of sodium cations. Subsequently, the affinity of CV molecules to be adsorbed onto the surface of SNPCs was enhanced. These results are compatible with the obtained results for the effect of pH, which revealed the main role of the electrostatic interaction in adsorption of the CV dye onto SNPCs-6 and SNPCs-8, while that onto SNPCs is less.

#### 3.2.3. Effect of Initial Concentration and Adsorption Isotherm Study

**Figure 9:**
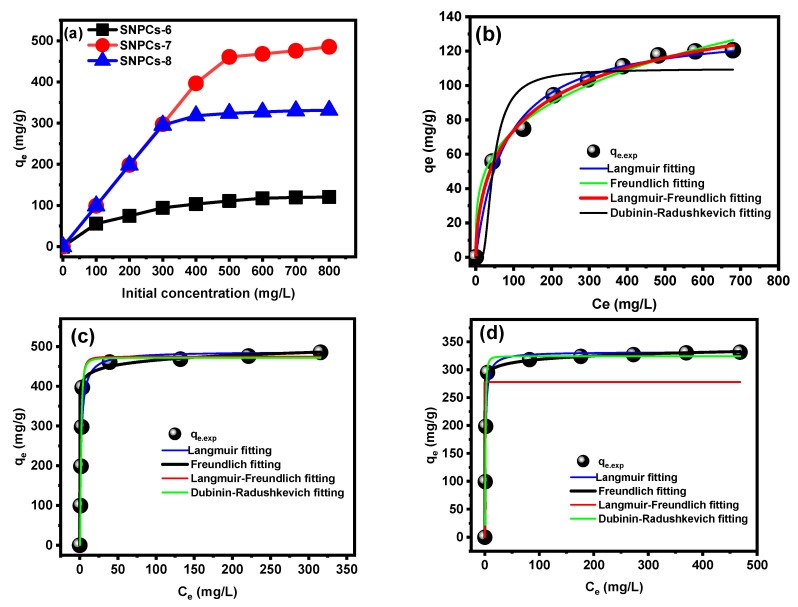
(**a**) Effect of initial concentration of CV on the adsorption process, and non-linear plots of various isotherm models for the adsorption of CV dye onto (**b**) SNPCs-6, (**c**) SNPCs-7 and (**d**) SNPCs-8.

The affinity between adsorbate and adsorbent can be interpreted by isotherm models, which is a helpful tool for describing the relationship between the residual concentration of the adsorbate in solution and its concentration on the surface of the adsorbent at a constant temperature. Currently, non-linear isotherm models like Langmuir [33], Freundlich [34], Langmuir–Freundlich [35], and Dubbinin–Radushkevich [36] were applied to study the adsorption behavior of CV dye onto the SNPCs. Supplementary Table S1 displays the equation and parameters of all used isotherm models. The relationship between the residual concentration of CV dye (*C_e_*, mg L^−1^) and the adsorption capacity (*q*_e_, mg g^−1^) of SNPCs and fitting of non-linear isotherm models are shown in Figure 9. The corresponding parameters of isotherm are listed in Table 2. To determine the best model to fit the equilibrium data and to describe the adsorption behavior, values of *R*^2^_red_ and *χ*^2^_red_ were calculated for each model. Low values of *χ*^2^_red_ and high values *R*^2^_red_ imply a high resemblance between the experiment and the model. Accordingly, the best isotherm model to describe the adsorption of the CV dye onto the surfaces of SNPCs-7 and SNPCs-8 followed Freundlich model while onto the surface of SNPCs-6 followed Langmuir-Freundlich model. Thus, it can be concluded that the multilayers of CV dye onto specific heterogeneous sites of the SPNCs surface occurred at equilibrium. The Dubbinin-Radushkevich model was used for calculating the adsorption energy (*E*, kJ mol^−1^), where the adsorption takes place physically at value less than 8 kJ mol^−1^, while the adsorption is chemically at higher value than 8 kJ mol^−1^. As listed in Table 2, the obtained result confirmed the physical adsorption process and agrees with the thermodynamic and desorption study. Thus, it can be concluded that the adsorption mechanism of CV onto SNPCs can be achieved by physical interaction such as electrostatic interaction, hydrogen bonding interaction, as well as π–π and n-π interaction. In addition, pore filling plays role in the adsorption process, especially on onto SNPCs-7. The maximum adsorption capacity (*Q*_max_, mg g^−1^) followed the order: SNPCs-7 (487.53 mg g^−1^) > SNPCs-8 (332.02 mg g^−1^) > SNPCs-6 (134.08 mg g^−1^), which agree with the order of the surface area and porosity.

#### 3.2.4. Effect of Contact Time and Kinetic Study

**Figure 10:**
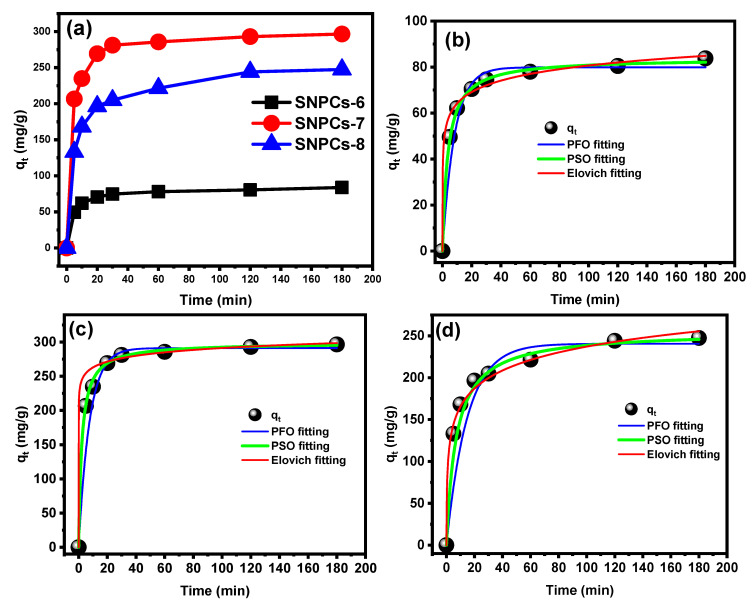
(**a**) Effect of contact time on the adsorption of CV dye, and non-linear plots of various kinetic models for the adsorption of CV dye onto (**b**) SNPCs-6, (**c**) SNPCs-7 and (**d**) SNPCs-8.

The mechanism and the rate of adsorption process can be interpreted in terms of kinetic parameters to show the efficiency of adsorbent materials. Figure 10 displays the effects of the contact time on the adsorption capacity of adsorbents towards the removal of CV dye. The adsorption capacity increased rapidly within the initial 20 min followed by slightly increase until the establishment of the equilibrium after 60 min. Within the first 5 min, 16.5%, 60.2%, and 44.3% were removed from the total CV dye concentration (300 mg L^−1^) by SNPCs-6, SNPCs-7, and SNPCs-8, respectively. This instantaneous phenomenon of adsorption indicated to the high affinity of SNPCs towards the adsorption of CV dye molecules. Currently, the adsorption kinetic of SNPCs-6, SNPCs-7, and SNPCs-8 were studied by non-linearized forms, namely pseudo-first-order (PFO) [37], pseudo-second-order (PSO) [38], and Elovich [39] models, as well as linearized forms of the intraparticle diffusion model, as listed in Table 3 and Supplementary Table S2, which displays the equation and parameters of all used kinetic models. According to the low values of nonlinear reduced chi-square statistics (*χ*^2^_red_) and high values of reduced determination coefficients (*R*^2^_red_), the best model for describing the adsorption kinetic of CV onto surface of SNPCs was the PSO model (*χ*^2^ = 13.81 − 0.817 and *R*^2^ = 0.9992 − 0.9998), compared to the PFO and Elovich models. Furthermore, the calculated value of the adsorption capacity (*q*_e,cal_.) was found to be much closer to the experimental value (*q*_e,exp_).

In porous materials, the adsorption kinetics by intraparticle diffusion model is an appropriate method for studying the mechanism for the transfer of dye from the surface to the pores. The two linear plots were obtained due to the varying extent of adsorption in the initial and final steps of adsorption experiment. The first step took place quickly and was assigned to the diffusion of CV from the solution to the external surface of SNPCs. The second step proceeded more slowly and was attributed to intraparticle diffusion effects. As shown in Supplementary Figure S2, the linear plot of SNPCs−6, SNPCs-7, and SNPCs-8 did not pass through the origin, confirming that the intraparticle diffusion was not only the rate-limiting step for the adsorption process but it was achieved simultaneously by more than one adsorption mechanism. Moreover, the values of Kid (1) for the intraparticle diffusion step are smaller than that of the film diffusion on the external surface. This result indicates that the transferring of CV dye from the external surface to pores is a gradual process.

#### 3.2.5. Effect of Temperature and Thermodynamic Studies

The effect of temperature on adsorption of adsorbate has a great role in describing the interaction between the adsorbent and adsorbate. The thermal experiments were performed at different temperatures (25, 35 and 45 °C), at concentration of SNPCs of 1 g L^−1^ and initial concentrations of CV (100, 400 and 600 mg L^−1^) at pH 10.0, respectively. The removal of CV by SNPCs-6, SNPCs-7, and SNPCs-8 increased from 60.5 mg g^−1^ to 98.2 mg g^−1^, 433.9 to 542.4 mg g^−1^, and 362.4 to 395.3 mg g^−1^ upon increasing the temperature from 25 to 45 °C, indicating the favorable removal CV at high temperatures (Supplementary Figure S3a). The increase in the adsorption capacity with temperature may be due to the decrease in the viscosity of CV dye, which facilitates the transfer of dye molecules from the outer surface into the pores of adsorbents. The obtained results agree with that obtained by Aichour [40] for the adsorption of CV dye by composite of activated bentonite/alginate.

The benefits of the adsorption process and the interaction mechanism between CV dye and adsorbent were investigated through the calculations of the thermodynamic parameters including free energy change (Δ*G*°), enthalpy (Δ*H*°), and entropy (Δ*S*°), using the following equations:

(7) $$\Delta G{}^{\circ}=-RTlnK_{c}$$

(8) $$lnK_{c}=\frac{\Delta S{}^{\circ}}{R}-\frac{\Delta H{}^{\circ}}{RT}$$

where *T* is the absolute temperature (K) and *R* is universal gas constant (8.3144 J mol^−1^ K^−1^). *K_c_* is the equilibrium constant that represents the ratio of the concentration of adsorbate on adsorbent (*C_ad_*) to the residual adsorbate concentration in solution at equilibrium state (*C_e_*). The values of *∆H°, ∆G*°, and ∆*S*° were calculated from the slope and intercept of the plot of ln*K*_c_ with respect to *1/T* (Supplementary Figure S3b), and presented results in Table 4.

According to the negative values of Δ*G*° in the temperature range of 25–45°C, the adsorption of CV dye onto SNPCs was feasible and spontaneous. Also, the increase in the absolute values of Δ*G*° at high temperatures indicated that the adsorption was favorable at higher temperatures. The positive values of ΔH° and ΔS° confirmed that the adsorption process was exothermic and the randomness increased at the solid–liquid interface, respectively, which was also supported by increasing the removal of CV with the increasing the temperature.

#### 3.2.6. Recycle Study

**Figure 11:**
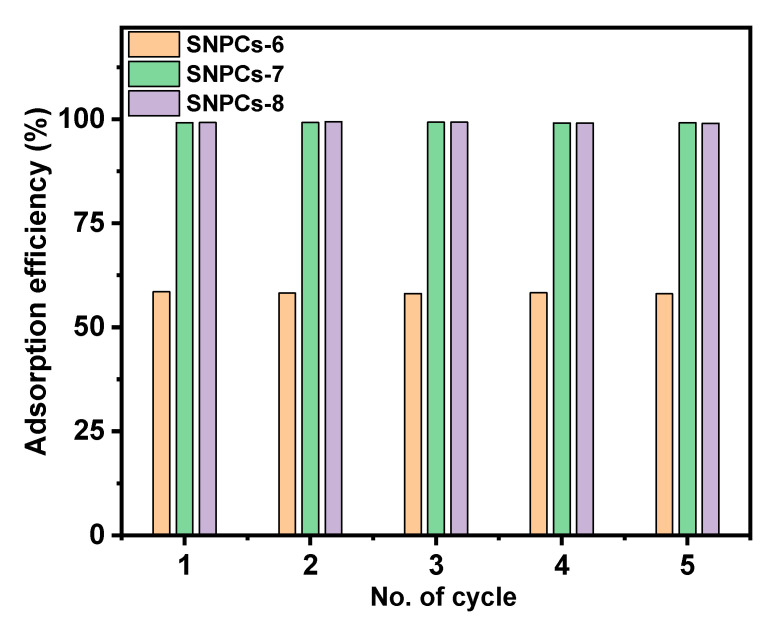
Reuse study of SNPCs.

The study of adsorption/desorption process plays an important role in clarifying the adsorption mechanism and the possibility of reusing the adsorbent and recovering the adsorbate. The reuse of adsorbent has a close relation with practical application in industries due to its cost depending on the regeneration process. In this work, the reusability and stability of the prepared adsorbents were investigated by subjecting them to five successive adsorption/desorption cycles. Figure 11 showed that the removal efficiency of all adsorbents was stable over five cycles. The adsorption efficiency after five cycles was found to be 58.05%, 99.15% and 99% for SNPCs-6, SNPCs-7, and SNPCs-8, respectively. It can be concluded that SNPCs can be used repeatedly without a loss of removal efficiency.

#### 3.2.7. Proposed Mechanism of Adsorption

**Figure 12:**
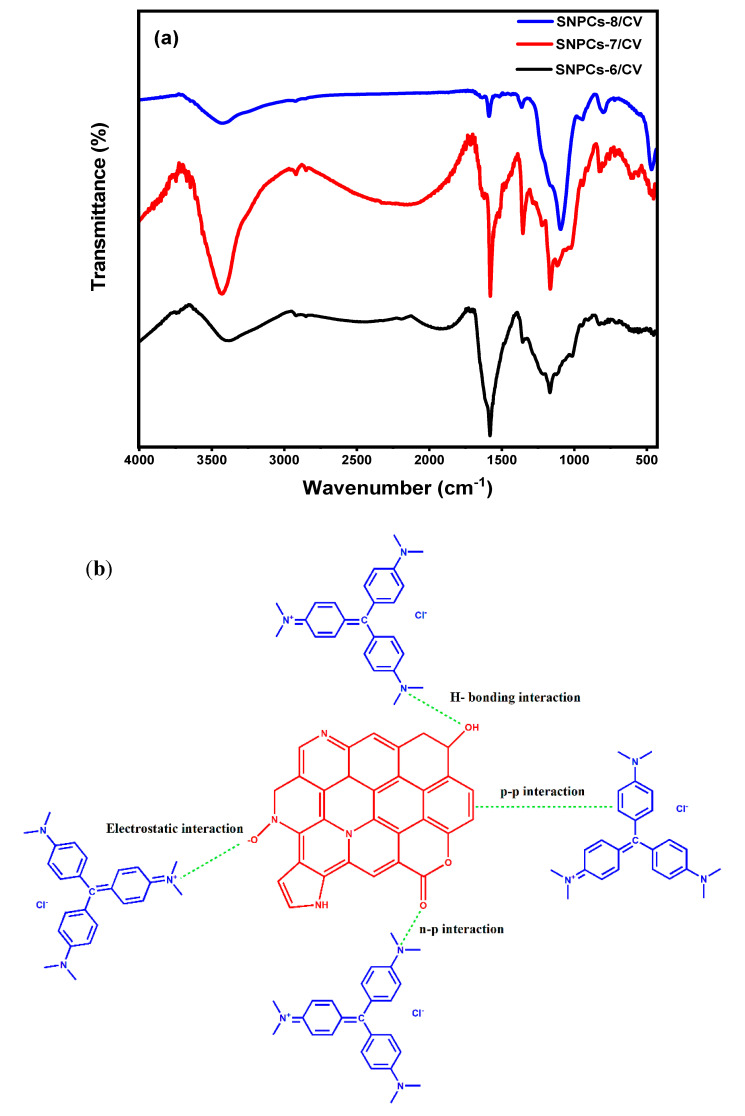
(**a**) FTIR of SNPCs after adsorption of CV dye and (**b**) the proposed adsorption mechanism of CV onto surface of SNPCs.

The proposed mechanism of adsorption can be proposed through the obtained results of the effect of pH, ionic strength, zeta potential measurement, as well as FTIR analysis after the adsorption of dye. On the basis of the effect of pH, ionic strength and zeta potential values, the adsorption capacity was varied which reflects the role of the electrostatic interaction between SNPCs and CV dye in the mechanism of adsorption. However, based on the FTIR of SNPCs after adsorption of dye, the hydrogen bonding is another role as well (Figure 12a). Figure 12a showed that the characteristic peaks of -OH bands were decreased and slightly shifted after adsorption, from 3445 to 3437 cm^−1^ for SNPCs-7 and 3436 to 3432 cm^−1^ for SNPCs-8. This result confirmed the formation of hydrogen bonding between the hydroxyl groups as H-donor on the surface of SNPCs-7 and SNPCs-8 and the nitrogen or oxygen atom as H-acceptor [41]. Similarly, slight shifting in the peaks of C=N and C=C groups and a decrease in the intensity also occurred, indicating that n-π and π–π interaction plays a role as well in the adsorption mechanism. Although oxygen containing functional groups on the surface of SNPCs-8 sample were higher than in SNPCs-7 and SNPCs-6, as confirmed by the EDX and FTIR results, the adsorption capacity of SNPCs-7 was higher than all, which clearly indicates that pore filling plays an important role in the mechanism of adsorption. Figure 12b showed the proposed adsorption mechanism of CV dye onto surface of SNPCs.

#### 3.2.8. Comparison with Other Adsorbents

Table 5 shows the adsorption capacity (*q*_e_, mg g^−1^) of SNPCs towards the removal of CV dye compared to other carbon materials and their composites. According to obtained results, it can be concluded that the SNPCs derived from P(1,5-DANPh) has remarkable higher adsorption capacity than all other carbon materials and their composites. This result reflects a promising future the importance of SNPCs materials for the removal of cationic dyes from wastewater.

## 4. Conclusions

SNPCs materials have been successfully prepared from P(1,5-DANPh) by single-step carbonization simultaneously with an alkali activation process. This approach represents a low-cost, efficient, and more productive method to obtain an effective adsorbent with high surface area and porosity. According to the results, the SNPCs-7 sample showed the highest surface area (1678.8 m^2^ g^−1^) with a micro-mesoporous structure. The isotherm study suggested that the best model for describing adsorption behavior was the Freundlich model. The maximum adsorption capacities at 25 °C were ranked as follows: SNPCs-7 (487.53 mg g^−1^) > SNPCs-8 (332 mg g^−1^) > SNPCs-6 (134.1 mg g^−1^). According to the kinetic study, the adsorption was fast, followed by the PSO model, and an equilibrium state could be reached after 60 min. Moreover, the percentage of dye removal was 44.3% by the SNPCs-7 sample during the first 5 min. The prepared SNPCs showed an excellent regeneration capacity in that it can be used more than five times in succession without a significant decrease in the adsorption efficiency. The effect of pH, desorption study, and FTIR analysis confirmed that the adsorption of CV dye onto the surface of SNPCs was reversible and controlled by more than one mechanism, including electrostatic interaction, π−π stacking, hydrogen bonding, and intraparticle diffusion. The thermodynamic parameters and mean energy values calculated from the D-R model indicated that the adsorption of CV dye on the SNPCs is a spontaneous, endothermic physisorption. Due to this simple and easy preparation method, unique properties, and high efficiency, SNPCs-7 is a promising, low-cost, and effective adsorbent for removing cationic dyes from contaminated water.

## Figures and Tables

**Table 1:** Textural characteristic properties of SNPCs samples.

Sample Code	SSA (m^2^ g^−1^)	V_total_ (cm^3^ g^−1^)	V_micro_ (cm^3^ g^−1^)	V_meso_ (cm^3^ g^−1^)	Meso Ratio (%)	A.V. Pore Width (nm)
SNPCs-6	857.20	0.489	0.360	0.129	26.38	2.28
SNPCs-7	1678.80	0.943	0.239	0.704	74.70	2.25
SNPCs-8	860.60	0.673	0.054	0.619	91.98	3.10

**Table 2:** Non-linear isotherm model of adsorption CV dye.

Model	Adsorbent		
	SNPCs-6	SNPCs-7	SNPCs-8
**Langmuir**			
*Q*_o_ (mg g^−1^)	134.08 ± 4.10	487.53 ± 21.03	332.03 ± 7.93
*K*_L_ (L mg^−1^)	0.0125 ± 0.0017	0.5558 ± 0.1135	0.7765 ± 0.1204
*R*_L_ (mg L^−1^)			
*R* ^2^_red_	0.9880	0.9518	0.9790
*χ* ^2^_red_	19.03	157.16	311.97
**Freundlich**			
*K*_F_ mg g^−1^/(mg L^−1^)^n^	20.26 ± 2.45	392.48 ± 12.90	279.76 ± 2.21
*1/n*	3.56 ± 0.2605	26.93 ± 4.69	35.59 ± 1.86
*R* ^2^_red_	0.9907	0.99975	0.9999
*χ* ^2^_red_	14.78	19.86	1.26
**Langmuir-Freundlich**			
*q*_max_ (mg g^−1^)	179.45 ± 32.29	474.64 ± 9.24	277.86 ± 32.37
*K*_L-F_ (L mg^−1^)	0.0055 ± 0.0035	0.5483 ± 0.0291	25.75 ± 0.00
*m*	0.5997 ± 0.1222	2.06 ± 0.2416	62.13 ± 0.00
*R* ^2^_red_	0.99518	0.9898	0.8390
*χ* ^2^_red_	7.67	340.18	8382.46
**Dubinin-Radushkevich**			
*q*_s_ (mg g^−1^)	109.61 ± 5.07	471.69 ± 11.11	324.05 ± 2.84
*K*_D-R_ (mol^2^ kJ^−2^)	264.73 ± 85.74	0.5544 ± 0.0557	0.3334 ± 0.0159
*E* (kJ mol^−1^)	0.0434	0.9497	1.22
*R* ^2^_red_	0.9012	0.9837	0.9968
*χ* ^2^_red_	157.16	540.94	46.94

**Table 3:** Non-linear kinetic model of adsorption CV dye.

Model	Adsorbent		
	SNPCs-6	SNPCs-7	SNPCs-8
**PFO**			
*q*_e,exp_	83.80	296.60	247.41
*q*_e,cal_	79.86 ± 1.49	284.28 ± 5.78	240.58 ± 5.25
*k*_1_	0.1207 ± 0.018	0.2253 ± 0.0262	0.0673 ± 0.0134
*R* ^2^_red_	0.9985	0.9836	0.9966
*χ* ^2^_red_	7.43	161.94	64.91
**PSO**			
*q*_t,cal_	83.80 ± 0.6698	298.96 ± 1.39	255.31 ± 3.72
*K*_2_	0.0032 ± 2.82 × 10^−4^	0.0015 ± 1.54 × 10^−4^	5.59 × 10^−4^ ± 8.58 × 10^−5^
*R* ^2^_red_	0.9998	0.9995	0.9992
*χ* ^2^_red_	0.8179	2.94	13.81
**Elovich**			
α	5572.83 ± 6790.98	2.13 × 10^9^ ± 1.02 × 10^10^	626.02 ± 260.91
*β*	0.1396 ± 0.0177	0.08094 ± 0.0175	0.0321 ± 0.0024
*R* ^2^_red_	0.9990	0.9993	0.9921
*χ* ^2^_red_	4.67	21.80	51.76
**Intraparticle diffusion**			
*K*_id(1)_	9.14 ± 1.98	27.96 ± 1.13	27.93 ± 4.49
*I*	30.65 ± 6.77	144.85 ± 3.84	74.00 ± 15.33
*R* ^2^_red_	0.9100	0.9968	0.9496
*K*_id(2)_	1.10 ± 0.0932	1.99 ± 0.1091	5.58 ± 0.9364
*I*	68.84 ± 0.9203	270.38 ± 1.08	177.05 ± 9.25
*R* ^2^_red_	0.9789	0.9910	0.9199

**Table 4:** Thermodynamic parameters for adsorption of CV dye onto SNPCs.

T (K)	Van’t Hoff Equation	*K*_c_	Δ*G*° (kJ mol^−1^)	Δ*H*° (kJ mol^−1^)	Δ*S*° (kJ mol^−1^)
**SNPCs-6**					
298	*y = –1672x + 54.52* *R*^2^ = 0.9453	0.43	−0.63	134.45	0.45
308		1.46	−5.16		
318		3.98	−9.69		
**SNPCs-7**					
298	*y = –5831.6x + 20.45* *R*^2^ = 0.9221	0.96	−2.17	48.48	0.17
308		1.28	−3.87		
318		2.24	−5.57		
**SNPCs-8**					
298	*y = 13382x + 46.29* *R*^2^ = 0.9662	1.49	−3.44	111.26	0.38
308		2.48	−7.28		
318		4.43	−11.13		

**Table 5:** Adsorption capacity of different carbon-based materials.

Adsorbent	*Q_o_* (mg g^−1^)	References
Fe_3_O_4_/Nitrogen-Doped Porous Carbon	457.12	[42]
Carbon produced from Eichhornia plant	50.50	[43]
Ag/activated carbon	87.2	[44]
SnFe_2_O_4_@activated carbon	158.73	[45]
Jute fiber carbon	27.99	[46]
Activated carbon derived from biomass	60.42	[47]
Magnetic activated carbon	44.7	[48]
MWCNTs/Mn_0.8_Zn_0.2_Fe_2_O_4_	5	[49]
Biochar derived from palm petiole	186	[50]
Woody biochar	125.5	[51]
Activated Charcoal	71.94	[52]
Oxidized MWCNTs	90.52	[53]
Self-nitrogen doped porous carbon (SNPCs)	487.53	This study

## Supplementary Materials

The following are available online at https://www.mdpi.com/2073-4360/12/11/2563/s1, Figure S1: Chemical and molecular 3d structure of CV dye, Figure S2: Intraparticle diffusion model of adsorption CV dye onto SNPCs, Figure S3: (a) Effect of temperature on adsorption of CV and (b) the plot between lnKd versus 1/T for obtaining the thermodynamic parameters Figure S4: SEM images of (a) SNPCs-6 (b) SNPCs-6 (c) SNPCs-6 after adsorption CV dye, Table S1. Description of adsorption isotherm models, Table S2. Description of adsorption kinetic models.
