# Supplementary material for: Self-Nitrogen-Doped Nanoporous Carbons Derived from Poly(1,5-diaminonaphthalene) for the Removal of Toxic Dye Pollutants from Wastewater: Non-Linear Isotherm and Kinetic Analysis
Source: Polymers (Basel). 2020 Oct 31;12(11):2563. doi: 10.3390/polym12112563 (PMC7693505; doi:10.3390/polym12112563)
Supplement: Supplementary file 1 [file polymers-12-02563-s001.pdf]

polymers

Self-Nitrogen-Doped Nanoporous Carbons Derived from Poly(1,5-diaminonaphthalene) for the Removal of Toxic Dye Pollutants from Wastewater: Non-linear Isotherm and Kinetic Analysis

Ali Aldalbahi *, Badr M. Thamer, Mostafizur Rahaman and Mohamed H. El-Newehy

Department of Chemistry, College of Science, King Saud University, Riyadh 11451, Saudi Arabia; aaldalbahi@ksu.edu.sa (A.A.); bthamer@ksu.edu.sa (B.M.T.); mrahaman1997@gmail.com (M.R.); melnewehy@ksu.edu.sa (M.H.E.)

* Correspondence: aaldalbahi@ksu.edu.sa

Received: 10 October 2020; Accepted: 28 October 2020; Published: date

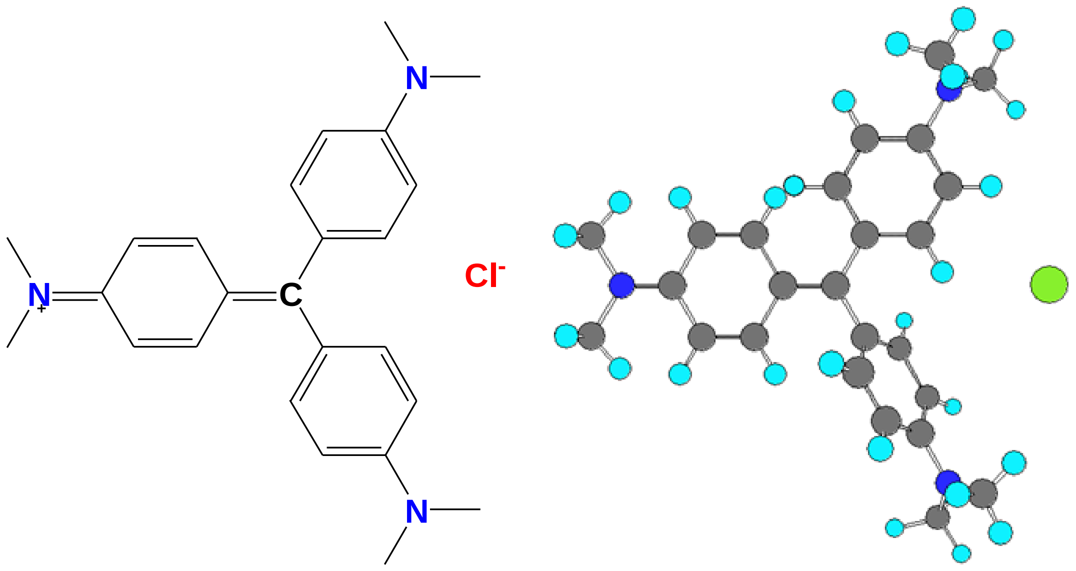

Figure S1: Chemical and molecular 3d structure of CV dye.

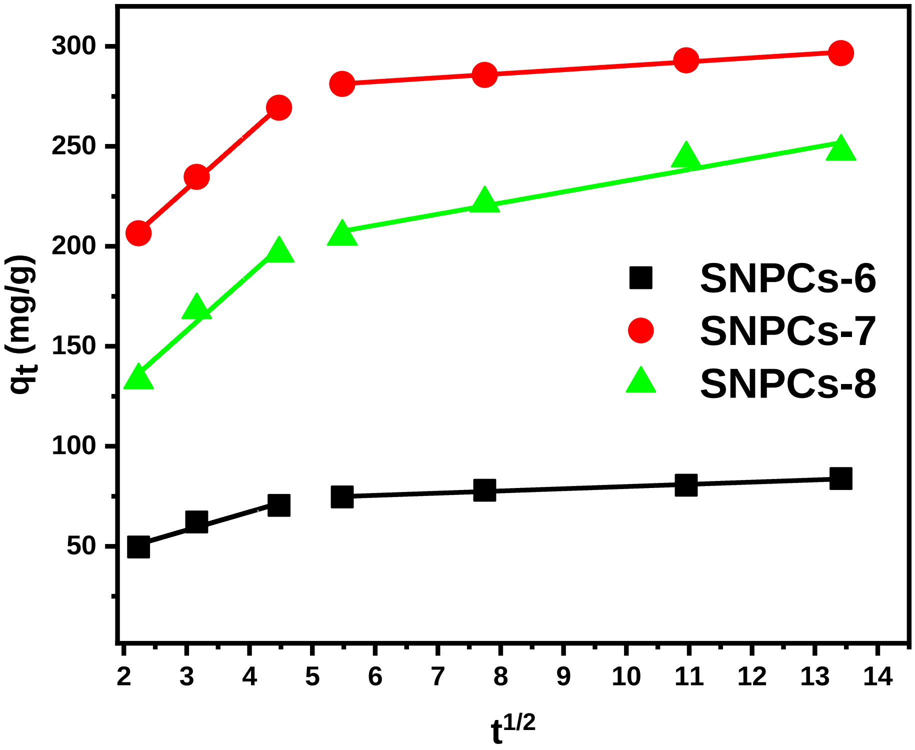

Figure S2: Intraparticle diffusion model of adsorption CV dye onto SNPCs.

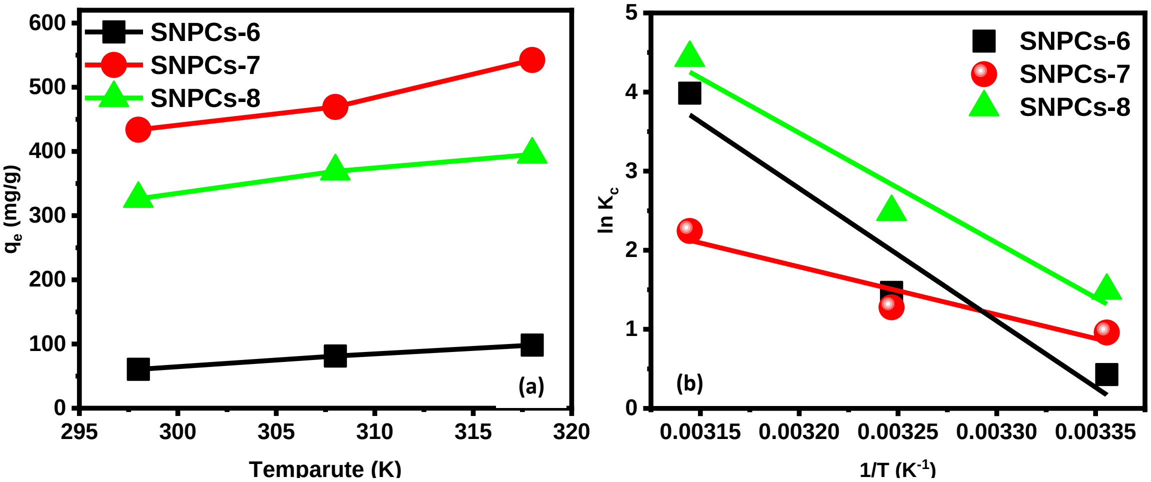

Figure S3: (a) Effect of temperature on adsorption of CV and (b) the plot between lnK_d versus $1/T$ for obtaining the thermodynamic parameters.

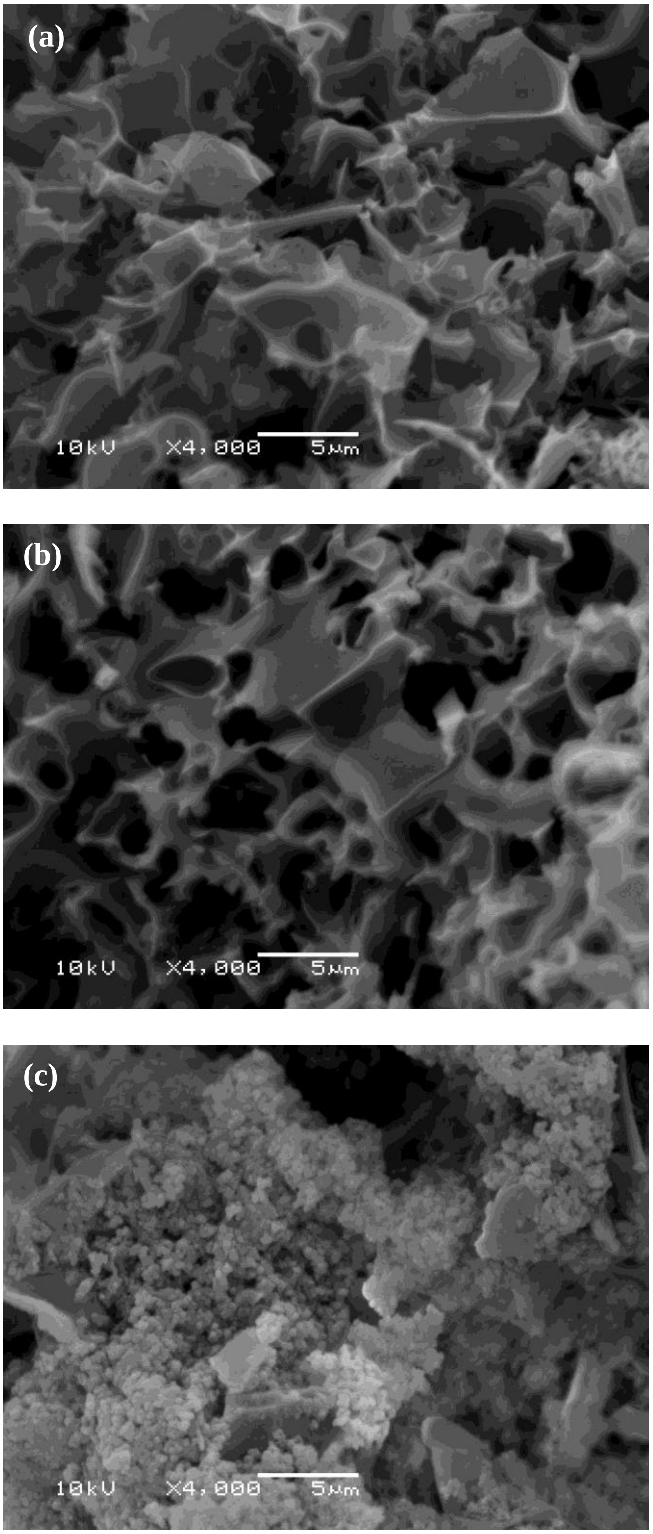

Figure S4: SEM images of (a) SNPCs-6 (b) SNPCs-6 (c) SNPCs-6 after adsorption CV dye.

polymers MDPI

Table S1. Description of adsorption isotherm models

Isotherm model	Equation	Parameters
**Langmuir**	$q_e = \frac{Q_o K_L C_e}{1 + K_L C_e}$	q_e (mg g^{-1}) = amount of dye adsorbed<br>C_e (mg L^{-1}) = dye concentration at equilibrium<br>Q_{omax} (mg g^{-1}) = maximum saturated monolayer adsorption capacity<br>K_L (L mg^{-1}) = Langmuir constant
**Freundlich**	$q_e = K_f\, C_e^{1/n}$	K_F [(mg g^{-1})/(L mg^{-1})^n] = Freundlich constant<br>n = Freundlich intensity parameter
**Langmuir-Freundlich**	$q_e = \frac{q_{max}\,(K_{LF} C_e)^m}{1 + (K_{LF} C_e)^m}$	$K_{L\text{-}F}$ (L mg^{-1}) = Langmuir-Freundlich constant<br>q_{max} (mg g-1): the maximum binding capacity<br>m: Langmuir-Freundlich isotherm exponen
**Dubinin-Radushkevich**	$q_e = q_o\, e^{-K_{D-R}\,\varepsilon^2}$<br>$\varepsilon = RTln(1 + \frac{1}{C_e})$<br>$E = \frac{1}{\sqrt{2K_{D-R}}}$	q_{DR} (mg g^{-1}) = adsorption capacity<br>K_{DR} (mol^2 kJ-^{2}) = constant related to the sorption energy<br>ε = Polanyi potential<br>E (kJ mol^{-1}) = mean adsorption energy<br>R (J $mol^{-1}K^{-1}$) = gas constant<br>T (K) = absolute temperature.

Note: If E is between 8 and 16 kJ mol^{-1}, adsorption is achieved by chemical processes, whereas when E < 8 kJ mol^{-1} physical processes dominate.

Table S2. Description of adsorption kinetic models

Kinetic model	Equation	Parameters
Pseudo-first-order	$q_t = q_e(1 - e^{-K_1 t})$	q_t = amounts of dye adsorbed at time t<br>q_e = amounts of dye adsorbed at equilibrium<br>K_1 (min-1) = rate constant of the PFO
Pseudo-second-order	$q_t = \frac{q_2^2 k_2 t}{1 + q_e K_2\, t}$	K_2 (g mg^{-1} min^{-1}) = rate constant of PSO
Elovich	$q_t = \frac{1}{\beta}\ln(1 + \alpha\beta t)$	α (mg g^{-1} min^{-1}) = initial adsorption rate<br>β (mg g^{-1}) = desorption constant during any one experiment
Intraparticle diffusion	$q_t = K_p\, t^{0.5} + C$	k_p (mg g^{-1} $min^{-1/2)}$ = represent the intraparticle diffusion rate constant<br>C (mg g_{-1}) = constant related to the thickness of the boundary layer
